# Dose regular physical activity improve the relationships among self-efficacy, resilience, happiness, and well-being in youth people with hearing disability in Guangdong—a moderation model

**DOI:** 10.3389/fpsyg.2025.1611657

**Published:** 2025-11-13

**Authors:** Wenpeng Zhan

**Affiliations:** School of Physical Education and Sports Science, Lingnan Normal University, Zhanjiang, Guangdong, China

**Keywords:** resilience, well-being, young people with hearing disabilities, regular physical activity, moderating effects

## Abstract

This study aims to explore the relationships among self-efficacy, resilience, happiness, and well-being in youth with hearing disabilities while also investigating the moderating effects of regular physical activity. The research used a quantitative method, random sampling was used to select 10 universities with disability support programs and purposive sampling was used to recruit eligible respondents. A total of 332 eligible respondents, university students diagnosed with moderate or greater hearing impairment, were included. Data analysis was conducted via SPSS 28.0 and SMART-PLS 4.0. The results revealed that self-efficacy significantly associates with happiness but not well-being, whereas resilience significantly associates with both happiness and well-being. Additionally, regular physical activity was found to moderate the relationships between self-efficacy and happiness, as well as between resilience and well-being. Notably, youth with hearing disabilities engaging in regular physical activity presented higher levels of self-efficacy, resilience, happiness, and well-being than did those not engaging in regular physical activity. The study emphasized the need to incorporate regular physical activity into the lives of young people with hearing disabilities to support their mental health and overall quality of life.

## 1 Introduction

Young people with disabilities face significant mental health challenges that can hinder their overall well-being and development. These challenges are worsened by factors such as social isolation, discrimination, and limited access to mental health services (World Health Organization, 2023). Sarkar’s (2023) research revealed that, compared with students without disabilities, students with disabilities experience more anxiety and academic-related distress, as well as higher rates of suicide ideation, suicide attempts, no suicidal self-injury, and similar problems. China has the highest number of individuals with hearing disabilities in the world. According to the China Disabled Persons’ Federation (2020), approximately 27.8 million people in China have hearing disabilities, representing over 30% of the country’s disabled population. Hearing disabilities have been identified as having severe negative effects on the health and quality of life of young people in China (Xiao et al., 2022). Young people with hearing disabilities are more likely to develop emotional and behavioral problems, which may lead to heightened stress (Xiao et al., 2022). Therefore, the mental health challenges faced by young people with hearing disabilities underscore the critical need for comprehensive mental health services and support within educational settings, a call for action that cannot be ignored.

Regular physical activity is a powerful tool for enhancing the mental health of young people with hearing disabilities. As per the World Health Organization (2020), this involves at least 150 min of moderate-intensity aerobic activity, 75 min of vigorous-intensity aerobic activity per week, or a combination of both. Research has shown that regular physical activity can effectively combat challenges such as social isolation and stigma by fostering social engagement and reducing feelings of loneliness (Schrempft et al., 2019; Franke et al., 2021). Moreover, it increases mental well-being, reduces symptoms of anxiety and depression, improves quality of life, and fosters social skills, resilience, and a sense of belonging (Porcelli et al., 2014; Charles and Chinaza, 2018) for young people with hearing disabilities. Therefore, regular physical activity is a health recommendation and a social lifeline for these young individuals.

The well-being and happiness of youth with hearing disabilities involve not only emotions but also powerful tools that can help them overcome challenges and improve their lives. Well-being refers to people’s affective and cognitive evaluations of their lives, typically operationalized as frequent positive affect, infrequent negative affect, and high life satisfaction (Diener, 1984). Happiness is a person’s overall positive emotional condition, marked by generally pleasant moods and feelings that substantially outweigh unpleasant ones (Haybron, 2008). Research has shown that these positive emotions can lead to better academic outcomes (Szarkowski and Brice, 2018), improved physical health (Veenhoven, 2008), enhanced social relationships (Camfield et al., 2009), and overall life satisfaction (Ruggeri et al., 2020). Additionally, they can boost the immune system, improve concentration and motivation, reduce the risk of chronic illness, and promote healthier lifestyles (Sin, 2016). For example, a study by McKenzie Smith et al. (2018) revealed that siblings of children with disabilities who reported high levels of well-being also showed better academic performance and social interactions. Therefore, well-being and happiness play crucial roles in the mental health and overall quality of life of youth with hearing disabilities. Understanding the factors that promote well-being and happiness is crucial, as this knowledge can help develop effective educational and social policies that can make a real difference in the lives of these youth.

Fostering self-efficacy can significantly improve the well-being and happiness of youth with hearing disabilities. These individuals often lack a sense of control over their lives, which can significantly impact their mental health and overall well-being (Palmer et al., 2017; Smetana, 2017). Self-efficacy gives them a sense of control and belief in their ability to positively influence outcomes, ultimately increasing their life satisfaction (Locke, 1997). Studies have demonstrated that self-efficacy can enhance a sense of control and competence (Locke, 1997; Patra, 2021), and importantly, it can reduce anxiety and stress (Kleppang et al., 2023), offering reassurance and confidence in the potential benefits. It also promotes positive social relationships (Caprara et al., 2006) and facilitates goal achievement (Meng and Zhang, 2023) for youth with hearing disabilities. For example, Patra (2021) reported that self-efficacy significantly correlates with and predicts the mental health and happiness of adolescents with hearing disabilities. These findings underscore the importance of nurturing self-efficacy to increase the well-being and happiness of youth with hearing disabilities.

Moreover, resilience plays a crucial role in helping youth with hearing disabilities handle various physical, social, and academic challenges (Luthar et al., 2000; Masten, 2018). Resilience, which involves adapting and recovering from adversity, trauma, or significant sources of stress, is essential for individuals to overcome difficulties and maintain their mental health and well-being (Luthar et al., 2000; Masten, 2018). Studies have shown that resilience contributes to emotional regulation (Compas et al., 2012), adaptability (Luthar et al., 2000), self-concept (Ragmoun and Alfalih, 2024), and social relationships (Cohen and Wills, 1985). Nesbitt et al. (2022) reported that resilience in transition-aged youth with serious mental illness was linked to reduced symptoms of anxiety and depression, as well as improved overall well-being. Therefore, developing resilience through strengthening psychological aspects can significantly improve the well-being and happiness of youth with hearing disabilities.

Regular physical activity is a crucial factor in connecting self-efficacy, resilience, happiness, and well-being, and it holds particular significance in improving the mental health of young people with hearing disabilities. From a neurological perspective, physical activity increases the levels of serotonin and dopamine, which are neurotransmitters that regulate mood and motivation and help prevent depression (Dishman et al., 2006; Pilc, 2010). The sense of achievement that comes with regular physical activity enhances psychological resilience and self-efficacy, contributing to overall well-being by reducing anxiety and depression (Pascoe et al., 2020). Additionally, it fosters positive social interactions and provides a structured routine that is essential for mental health, offering a sense of security and stability (Schuch and Vancampfort, 2021). According to Yang et al. (2022), the use of the RE-AIM framework to analyze the impact of physical activity on mental health outcomes revealed significant improvements in psychological health, including reduced anxiety and depression, and enhanced self-esteem and cognitive function. Beyond psychosocial determinants, recent educational data-mining work shows that student performance can be modeled from socio-academic features using modern machine-learning pipelines. For example, Al-Ali et al. (2024) analyzed socio-academic factors with EDA, dimensionality reduction, clustering, and sequence models to predict academic performance, illustrating how data-driven approaches uncover multivariate patterns not easily captured by traditional analyses.

**Figure 1:**
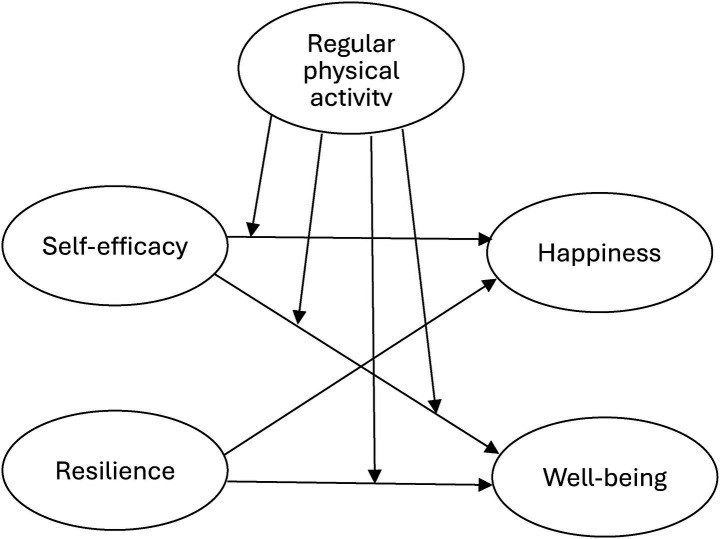
Research framework.

In conclusion, while there is extensive evidence of the benefits of regular physical activity for mental health, previous studies have not thoroughly investigated its effects on the connections among self-efficacy, resilience, happiness, and overall well-being in young people with hearing disabilities. This study aims to achieve two objectives: (1) identify the relationships among self-efficacy, resilience, happiness, and well-being from the perspective of young people with hearing disabilities and (2) examine the associates with and moderating effects of regular physical activity on these relationships (Figure 1).

## 2 Methods

### 2.1 Research design

This study aims to explore the relationships among self-efficacy, resilience, happiness, and well-being among young people with disabilities, as well as the moderating effect of regular physical activity on mental health. A quantitative method and cross-sectional method were employed to address these issues. The questionnaire used in the study was developed on the basis of previous related studies. It was reviewed by three professors with experience in the mental health of young people with disabilities to ensure content validity. The eligible respondents were university students in mainland China with hearing disabilities. Teachers assisted in distributing the questionnaires to the respondents. The reliability and validity of the scales and structured model were analyzed via partial least squares structural equation modeling (PLS-SEM). Additionally, an independent sample t test was used to analyze the effects of regular physical activity on these relationships.

### 2.2 Research instruments

The research instruments used in this study are divided into five sections. The first section includes a self-efficacy scale based on the scale developed by Crowe (2021), specifically for individuals with hearing disabilities, consisting of 10 items. The second section encompasses the resilience scale, drawn from the scale developed by Radovanović et al. (2020) for deaf and hard-of-hearing adolescents. This resilience scale is divided into three dimensions—sense of mastery (three items), sense of relatedness (three items), and emotional reactivity (three items)—for a total of nine items. The third section contains the happiness scale, adapted from the scale developed by Ruksana et al. (2023) for deaf and hard-of-hearing individuals, which includes four items. The fourth section involves the well-being scale, which is based on the work of Ban et al. (2024), is designed explicitly for deaf students and contains four significant items. All these scales utilize a seven-point Likert scale, with response options ranging from “strongly agree” to “strongly disagree,” assigning scores from seven to one, respectively. The final section of the questionnaire is dedicated to collecting respondents’ demographic information and physical activity behavior in a thorough and comprehensive manner, including gender, academic year, degree of hearing impairment, type of hearing impairment, regular physical activity habits, and types of physical activities.

### 2.3 Data collection

Data collection was conducted by two phrases. First, this study involved randomly selecting 10 universities in Guangdong Province (78 universities) that offer programs for students with disabilities. Second, purposive sampling was used to select eligible respondents who were university students diagnosed with moderate or more significant hearing impairment (bilateral hearing loss of 41–60 dBHL or above) according to the World Health Organization’s (2007) guidelines (World Health Organization, 2007). To gather data, the researcher sent an invitation email to the teachers in charge of the programs, inviting them and their students to participate in a questionnaire survey. After receiving permission, the researcher distributed hard-copy questionnaires on site, with the teachers assisting in translating and communicating with the students to ensure that each student understood the purpose and significance of the study. The questionnaire survey was conducted anonymously, and the purpose of the study, respondents’ rights, and data usage were explained in detail before the students filled out the questionnaire. Data collection took place from December 2023 to April 2024, ensuring a comprehensive and thorough process. A total of 354 questionnaires were collected, with 22 excluded because of incomplete responses, resulting in 332 valid questionnaires.

The demographic information collected from the respondents indicates that 332 of them were predominantly male (54.5%) and first-year students (27.4%). The majority reported a moderate level of hearing impairment (56.9%), with genetics (28.9%) and trauma (29.8%) being the main causes. Notably, aerobic exercise was the most favored type of physical activity among the participants (87.7%), and a significant number of them adhered to a regular exercise routine (66.6%) (Table 1).

**Table 1:** The respondents’ demographic information.

Variables	Frequency	Percentage (%)
Gender		
Male	181	54.5
Female	151	45.5
Grade		
Freshman	91	27.4
Sophomore	81	24.4
Junior	87	26.2
Senior	73	22.0
Grade of hearing impairment		
Moderate impairment	189	56.9
Severe impairment	80	24.1
Profound impairment	63	19.0
Causes		
Genetics	96	28.9
Ear infection	36	10.8
Chemical exposure	37	11.1
Long-term noise exposure	64	19.3
Trauma	99	29.8
Type of exercise		
Aerobic exercise	291	87.7
Anaerobic exercise	31	9.3
Both	10	3.0
Regular exercise habit		
Yes	221	66.6
No	111	33.4

### 2.4 Data analysis

The collected data were analyzed via SPSS 28.0 and SMART-PLS 4.0. Independent samples t tests were conducted to examine the impact of regular physical activity on self-efficacy, resilience, happiness, and well-being. CFA was used to evaluate the scales’ reliability and validity. Finally, structural equation modeling (SEM) was employed to confirm the connections between self-efficacy, resilience, happiness, and well-being and to examine the moderating effect of regular physical activity.

### 2.5 Ethical considerations

This study, approved by the Academic Committee of the School of Physical Science at Lingnan Normal University (Approval No. LNU 2023601), ensured that informed consent was obtained from the respondents through a two-stage process. The teachers and researchers who assisted in the questionnaire distribution thoroughly explained the study’s purpose and the participants’ rights, emphasizing the protection of their rights. After providing informed consent, the respondents were asked to fill out the questionnaire. The first page of the questionnaire included a detailed informed consent form, and the respondents were asked to read the content to ensure that their rights were protected.

## 3 Results

### 3.1 Reliability and validity

The present study meets the standard requirements for reliability and validity (see Table 2). The self-efficacy items (SE1 to SE10) exhibit factor loadings ranging from 0.639 to 0.887, indicating a moderate to high correlation with the underlying construct. The Cronbach’s α for self-efficacy is 0.931, indicating excellent internal consistency. The C. R. is 0.941, further confirming the reliability. The AVE is 0.619, indicating that the construct captures over 61% of the variance, providing comprehensive coverage of the self-efficacy construct. The resilience scale also demonstrated excellent reliability and validity. The high factor loadings range from 0.603 to 0.881, indicating robust correlations with the underlying construct. The Cronbach’s α is 0.932, indicating excellent internal consistency. The C. R. is 0.944, further confirming the scale’s reliability, with an AVE of 0.654, suggesting that the construct captures a significant portion of the variance. Additionally, the happiness-related items (H1–H4) have factor loadings ranging from 0.852 to 0.956, indicating strong correlations with the underlying construct. The Cronbach’s α is 0.940, and the CR is 0.957, indicating excellent reliability. The AVE is 0.848, which suggests that the construct explains 84.8% of the variance. Finally, the well-being items (WB1 to WB4) have very high factor loadings, ranging from 0.881 to 0.939. The Cronbach’s α is 0.940, and the CR is 0.957, with an AVE of 0.849, indicating high reliability and validity.

**Table 2:** Reliability and validity.

Variable	Dimensions	Item	Factor loading	Cronbach’s α	C. R.	AVE
Self-efficacy	–	SE1	0.860	0.931	0.941	0.619
		SE2	0.815			
		SE3	0.856			
		SE4	0.867			
		SE5	0.877			
		SE6	0.887			
		SE7	0.639			
		SE8	0.648			
		SE9	0.639			
		SE10	0.712			
Resilience	Sense of mastery	SM1	0.881	0.932	0.944	0.654
		SM2	0.875			
		SM3	0.875			
	Sense of relatedness	SR4	0.847			
		SR5	0.798			
		SR6	0.603			
	Emotional reactivity	ER7	0.790			
		ER8	0.714			
		ER9	0.851			
Happiness	–	H1	0.952	0.940	0.957	0.848
		H2	0.852			
		H3	0.956			
		H4	0.919			
Well-being	–	WB1	0.933	0.940	0.957	0.849
		WB2	0.939			
		WB3	0.881			
		WB4	0.929			
	Goodness of fit				0.756	

In partial least squares analysis, goodness-of-fit (GoF) is an essential indicator of model fit. The GoF value indicates how well the model explains the observed data. Akter et al. (2011) state that a higher GoF reflects greater explanatory power of the model’s estimated parameters.


$$\mathrm{GoF}=\sqrt{\text{average}\;\mathrm{AVE}\times \text{average}\;R^{2}}$$

Akter et al. (2011) proposed that values for goodness of fit (GoF) can be categorized into three ranges: above 0.36 indicates a high level of model fit, 0.25–0.35 indicates a moderate level of model fit, and 0.10–0.24 indicates an acceptable level of model fit. The GoF value for the model in this study is 0.756, indicating a solid fit (Table 2).

### 3.2 Structured model analysis

**Figure 2:**
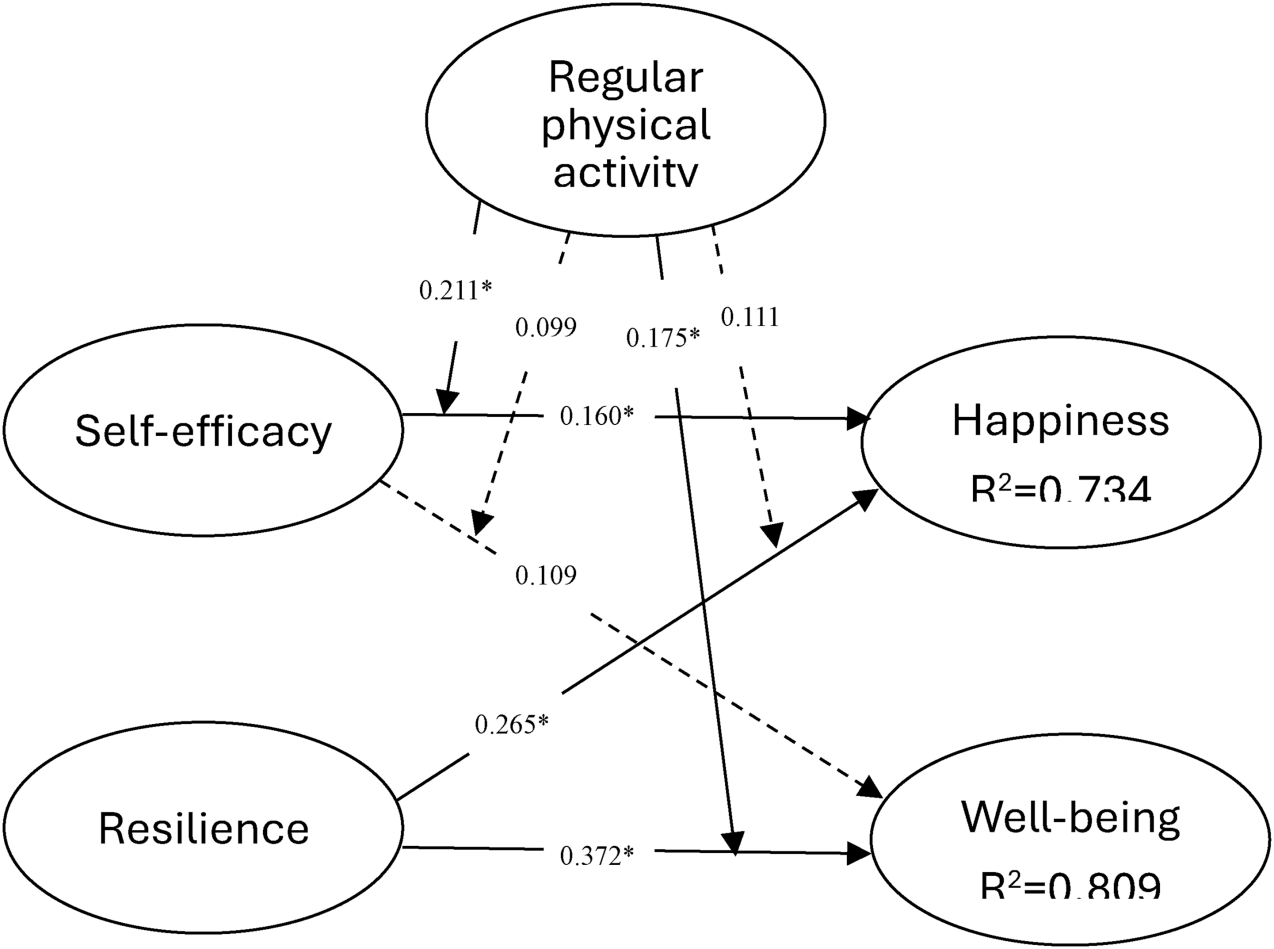
Results of the model.

Research has indicated that self-efficacy among young people with hearing disabilities significantly associates with their happiness (β = 0.160*; *p* < 0.05). This finding suggests that having stronger self-efficacy beliefs can enhance their happiness. However, self-efficacy does not significantly affect overall well-being (β = 0.109; *p* > 0.05), implying that self-efficacy may not improve the overall well-being of young people with hearing disabilities. On the other hand, resilience among young people with hearing disabilities significantly associates with both their happiness (β = 0.265; *p* < 0.05) and well-being (β = 0.372; *p* < 0.05). This finding indicates that greater resilience is a reassuring factor that strongly supports happiness and well-being (Figure 2).

**Figure 3:**
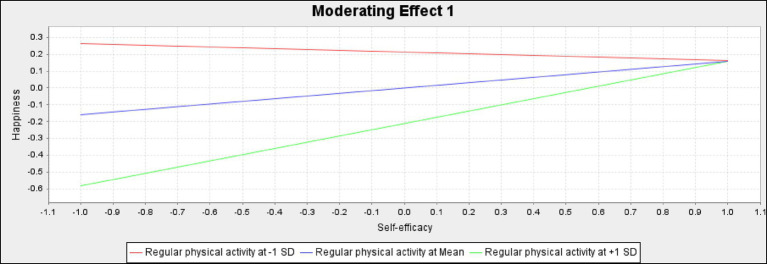
Moderating effects of regular physical activity on the relationship between self-efficacy and happiness.

**Figure 4:**
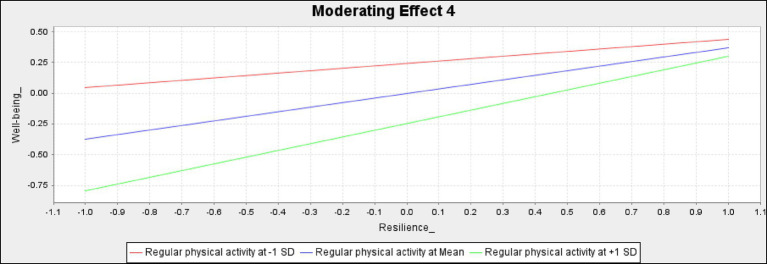
Moderating effects of regular physical activity between resilience and well- being.

The effects of regular physical activity on young people with hearing disabilities are noteworthy. Our research findings strongly support the importance of regular physical activity in promoting the well-being of these individuals. We found that regular physical activity significantly associates with the relationships between self-efficacy and happiness (β = 0.211*; *p* < 0.05), as well as between resilience and well-being (β = 0.175*; *p* < 0.05). According to the slope analysis (Figures 3, 4), regular physical activity amplifies the positive association between self-efficacy and happiness. In other words, higher levels of physical activity strengthen the link between self-efficacy and happiness, whereas lower levels weaken this relationship. Additionally, regular physical activity enhances the positive association of resilience on well-being, with higher levels of physical activity bolstering the benefits of resilience and lower levels diminishing the relationship. These findings underscore the importance of regular physical activity for promoting well-being in young people with hearing disabilities and should convince us all to commit to integrating physical activity into their daily lives.

### 3.3 T test analysis

The study results show that young people with hearing disabilities who regularly engage in physical activity demonstrate significantly higher levels of self-efficacy (*t* = 15.956*; *p* < 0.05), resilience (*t* = 16.645*; *p* < 0.05), happiness (*t* = 13.143*; *p* < 0.05), and well-being (*t* = 15.388*; *p* < 0.05) than their counterparts who do not engage in regular physical activity. These findings confirm that regular physical activity is beneficial for enhancing the mental health of young people with hearing disabilities (see Table 3).

**Table 3:** Results of the *t*-test.

Dependent variables	Independent variables	*M*	SD	*t* values
Self-efficacy	Regular physical activity	6.679	0.500	15.956*
	Nonregular physical activity	5.133	0.957	
Resilience	Regular physical activity	6.692	0.435	16.645*
	Nonregular physical activity	5.166	0.915	
Happiness	Regular physical activity	6.933	0.219	13.143*
	Nonregular physical activity	5.658	1.011	
Well-being	Regular physical activity	6.927	0.218	15.388*
	Nonregular physical activity	5.491	0.971	

*p < 0.05.

## 4 Discussion

A recent study revealed several significant findings regarding young people with hearing disabilities. First, it has been shown that self-efficacy has a positive effect on happiness, which is supported by the findings of Palmer et al. (2017), Patra (2021), and Smetana (2017). Self-efficacy can help empower young people with hearing disabilities to effectively manage the challenges they face due to their condition, giving them greater control over their lives (Michael et al., 2015). This sense of control is linked to reduced anxiety and stress, improved social interactions, and a more positive outlook, all of which contribute to increased happiness (Patra, 2021). Compared with their hearing peers, young people with hearing disabilities often have lower levels of self-efficacy (Cuevas et al., 2019). When they lack confidence in managing their lives and dealing with various challenges, anxiety and excessive stress can occur, ultimately affecting their mental health. Therefore, enhancing self-efficacy among young people with hearing disabilities is a critical first step toward promoting their happiness.

Additionally, our study revealed that regular physical activity has significant potential to moderate the link between self-efficacy and happiness. Young people with hearing disabilities who regularly engage in physical activity demonstrate a stronger connection between self-efficacy and happiness. From a physiological standpoint, Gerber (2018) suggested that positive changes in hypothalamic–pituitary-adrenocortical axis activity and the sympathoadrenal medullary system during regular exercise can extend to nonphysical stressors. Multiple studies have demonstrated that regular physical activity not only directly contributes to better health by reducing the risk factors for major diseases but also indirectly helps individuals manage stress and enhance self-efficacy (McAuley et al., 2005; Gerber and Pühse, 2009; Gerber, 2018). Therefore, establishing a regular exercise routine among young people with hearing disabilities holds promising potential to significantly increase their confidence and mental well-being, offering a hopeful path for their future.

Surprisingly, our study revealed no significant relationship between self-efficacy and well-being among young people with hearing disabilities. Enhancing self-efficacy may not necessarily lead to improved well-being for these individuals. This finding diverges from those of previous research, which consistently demonstrated a positive correlation between self-efficacy and well-being (Oyewumi and Anieke, 2016; Crowe, 2021; Muñoz et al., 2021). Several studies have attempted to explain why significant relationships are absent. Gross (2002) proposed that while self-efficacy can equip individuals to confront challenges, a deficiency in emotional regulation skills or resilience might impede their well-being. Furthermore, given the intricate nature of well-being, self-efficacy alone may not be adequate to foster well-being, especially when it is centered on a belief in one’s ability to succeed (Diener, 2000). Furthermore, In collectivist, face-saving contexts, perceived capability may not translate into higher well-being when autonomy, inclusion, and accessible supports are constrained; stigma and service/access barriers can blunt the affective returns of self-efficacy (Corrigan and Watson, 2002). Therefore, the use of self-efficacy as the sole determinant for promoting well-being in young people with hearing disabilities may be constrained. These findings underscore the need for further research to better understand and promote the well-being of the population.

Resilience has a significant positive effect on the happiness and well-being of young people with hearing disabilities. This finding is supported by previous studies, which have shown that resilience plays a crucial role in promoting mental health and enhancing happiness (Marwaha and Anand, 2019; Patra, 2021) and well-being (Porcelli et al., 2014; Conder et al., 2015; Patra, 2021). Resilience refers to the ability of individuals to use adaptive coping strategies to effectively manage stress and overcome challenges, leading to positive mental health outcomes (Bonanno, 2004). A critical component of resilience that contributes to happiness and well-being is psychological flexibility, which enables individuals to adapt to changing circumstances and see challenges as opportunities for growth (Kashdan and Rottenberg, 2010). Therefore, resilience is crucial for young people with hearing disabilities, as it helps them adapt to difficult situations and cope with the stress associated with life challenges, ultimately fostering a positive outlook essential for maintaining mental health.

This study suggests that regular physical activity plays a role in moderating the relationship between resilience and well-being. These findings indicate that young people with hearing disabilities who engage in regular physical activity exhibit greater resilience, improving their well-being. This finding aligns with several previous studies that have demonstrated the positive impact of regular physical activity on resilience and well-being (Reyes-Molina et al., 2022; Wermelinger Ávila et al., 2022). According to Liu et al. (2024), maintaining a regular physical activity routine can help individuals become more mentally resilient, benefiting their mental well-being. Therefore, young individuals with hearing disabilities need regular physical activity to develop resilience and enhance their well-being.

Finally, this study revealed that young people with hearing disabilities who engage in regular physical activity tend to have higher levels of self-efficacy, resilience, happiness, and well-being than those who do not engage in regular physical activity. These findings are consistent with previous research that has shown the positive effects of regular physical activity on self-efficacy (Toros et al., 2023), resilience (Deshayes and Périard, 2023), happiness (Liang et al., 2021), and well-being (Herbert et al., 2020). Engaging in regular physical activity has been found to improve cognitive function, reduce symptoms of depression and anxiety, and enhance overall psychological well-being due to neurochemical changes, such as increased production of endorphins and serotonin, associated with consistent exercise (Miller et al., 2016; McPherson et al., 2018). Young people with hearing disabilities often face high-stress situations in their daily lives due to limitations in their body function. They require effective strategies to manage this stress. This study confirms that regular physical activity can significantly improve self-efficacy, resilience, happiness, and well-being in young people with hearing disabilities, contributing to the development of their mental health.

## 5 Theoretical contributions and implications

The findings of this study have several theoretical contributions and implications.

### 5.1 Theoretical contributions

The main theoretical contribution of this study is the understanding of how regular physical activity affects the relationships among self-efficacy, happiness, and resilience in young people with hearing disabilities. While previous studies have shown that regular physical activity can promote mental health, this study addresses the lack of model validation and a specific focus on special populations. Additionally, contrary to most studies that support a positive relationship between self-efficacy and well-being, this study revealed that self-efficacy did not significantly associates with well-being in young people with hearing disabilities. This finding requires further exploration in future studies. Finally, the study suggests that resilience is critical in promoting happiness and well-being in young people with hearing disabilities. Building resilience to adapt to daily environments and cope with challenges may be more important than enhancing self-efficacy.

### 5.2 Implications

The study findings have two practical implications. First, it is crucial to build resilience in young people with hearing disabilities as a priority step toward achieving mental health. Lindsay (2011) suggested that young people with hearing disabilities can develop resilience and lead more empowered lives by combining self-advocacy training, life skills development, and goal setting. In addition to providing timely support and assistance, schools and families should focus on cultivating independent living skills to increase adaptability. Second, colleges in mainland China have actively developed policies to strengthen physical education, emphasizing the importance of students cultivating exercise habits for their physical and mental well-being (The State Council, 2020). However, there seems to be a lack of initiatives specifically promoting regular physical activity for young people with hearing disabilities or other special populations. Schuch and Vancampfort (2021) suggest that universities should implement structured, sign-language–accessible physical activity programs (captioned classes and sports adapted for Deaf and hard-of-hearing students), mentorship and peer-buddy systems, and inclusive, credit-bearing physical education designed with universal design for learning principles, supported by accessible environments with clear visual cues and vibration timers, staff training in Deaf culture and visual coaching, behavior-change supports such as specific, measurable, attainable, relevant, and time-bound goals with self-monitoring, integrated referrals between counseling services and campus recreation, and monitoring based on reach, effectiveness, adoption, implementation, and maintenance to sustain participation. Given the proven benefits of regular physical activity on the mental health of young people with hearing disabilities, it is essential for the government and colleges to proactively implement policies that focus on enhancing physical education for special populations and helping them develop regular exercise habits. For example, university offers sign-language–accessible group classes and sports adapted for Deaf and hard-of-hearing students (for example, swimming, track, small-sided football), with on-site interpreters, captioned materials, clear visual demonstrations, and vibration timers. Government can promote that integrate referral pathways between student health services, counseling, and campus recreation so that students with elevated stress or anxiety receive structured activity prescriptions and follow-up.

## 6 Limitations of the study

This study used rigorous methods to achieve its objectives. However, several limitations still need to be addressed. First, the study’s cross-sectional design captures data only at a specific point in time. A longitudinal approach would help establish cause–and–effect relationships between the variables in future studies. Second, the study focused only on young people with hearing disabilities, excluding others with disabilities. Future studies could consider including a broader range of disabilities to provide more comprehensive insights. Additionally, the study primarily used quantitative methods, which may not capture the depth of individual experiences. The use of qualitative methods could provide richer insights into how young people with hearing disabilities perceive and build resilience and self-efficacy in their daily lives and how regular physical activity benefits their mental health and overall well-being.

## 7 Conclusion

This study revealed that regular physical activity significantly improved self-efficacy, resilience, happiness, and well-being among young people with hearing disabilities. The findings emphasize the vital role of regular physical activity in promoting mental health, especially by strengthening resilience, which is crucial for coping with the unique challenges faced by this population. Additionally, the study reveals that while self-efficacy positively associates with happiness, it does not directly affect well-being, suggesting that other factors, such as emotional regulation, play a more significant role in the development of well-being. Overall, this study highlights the importance of including regular physical activity in young people with hearing disabilities to support their mental health and overall quality of life.

## Data Availability

The raw data supporting the conclusions of this article will be made available by the authors, without undue reservation.
