# Supplementary material for: Dose regular physical activity improve the relationships among self-efficacy, resilience, happiness, and well-being in youth people with hearing disability in Guangdong—a moderation model
Source: Front Psychol. 2025 Nov 13;16:1611657. doi: 10.3389/fpsyg.2025.1611657 (PMC12657399; doi:10.3389/fpsyg.2025.1611657)
Supplement: Supplementary file 1 [file Data_Sheet_1.pdf]

Table 1 The items of the scale

Variables	Items	References
Self-efficacy	1. I can always manage to solve difficult problems if I try hard enough.	Crowe (34)
	2. If someone opposes me, I can find the means and ways to get what I want.	
	3. It is easy for me to stick to my aims and accomplish my goals.	
	4. I am confident that I could deal efficiently with unexpected events.	
	5. Thanks to my resourcefulness, I know how to handle unforeseen situations.	
	6. I can solve most problems if I invest the necessary effort.	
	7. I can remain calm when facing difficulties because I can rely on my coping abilities.	
	8. When I am confronted with a problem, I can usually find several solutions.	
	9. If I am in trouble, I can usually think of a solution.	
	10. I can usually handle whatever comes my way.	
Resilience	1. I believe that positive things will happen in my future.	Radovanović et al. (35)
	2. I am confident in my ability to make a difference in the outcomes of my life.	
	3. I can adjust easily when circumstances in my life change.	
	4. I feel secure in my relationships with others.	
	5. I believe that I have people in my life who will support me when I need help.	
	6. I feel comfortable and at ease when I am with other people.	
	7. I tend to have strong emotional responses to things that happen in my life.	
	8. I can quickly recover from emotional setbacks and return to my usual self.	
	9. My emotions sometimes interfere with my ability to function in daily life.	
Happiness	1. In general, I consider myself a happy person.	Ruksana et al. (36)
	2. Compared to most of my peers, I feel happier.	
	3. I am satisfied with the way my life is going.	
	4. I feel that my life is full of rewarding experiences.	
Well-being	1. I am satisfied with my academic performance.	Ban et al. (37)
	2. I am satisfied with my current life.	
	3. I have good relationships with my peers.	
	4. I feel positive about who I am as a person.	
